# Hydrocortisone 21-hemisuccinate did not prevent exogenous GAPDH-induced apoptosis in human neuroblastoma cells

**DOI:** 10.1016/j.dib.2018.08.093

**Published:** 2018-08-30

**Authors:** Vladimir F. Lazarev, Elizaveta A. Dutysheva, Elena Y. Komarova, Irina V. Guzhova, Boris A. Margulis

**Affiliations:** Institute of Cytology of Russian Academy of Science, Tikhoretsky pr. 4, St-Petersburg 194064, Russia

## Abstract

These data are related to our paper “GAPDH-targeted therapy – a new approach for secondary damage after traumatic brain injury on rats” (Lazarev et al., In press), in which we explore the role of exogenous GAPDH in traumatic brain injury-induced neuron death, and the therapeutic application of small molecules that bind to the enzyme. The current article demonstrates the induction of apoptosis by exogenous GAPDH and the effectiveness of the hydrocortisone derivative for suppressing the pathogenic action of the enzyme.

**Specifications Table**

**Table:** 

Subject area	*Biology*
More specific subject area	*Cell biology*
Type of data	*Text file, graph*
How data was acquired	*Ultrafiltration, acridine orange staining*
Data format	*Analysed*
Experimental factors	*Pure GAPDH and hydrocortisone derivative were used in in vitro experiments*
Experimental features	*Exogenous pre-denatured GAPDH induces apoptosis in neuroblastoma SH-SY5Y cells and hydrocortisone derivative did not prevent GAPDH-induced apoptosis in vitro.*
Data source location	*St. Petersburg, Russia*
Data accessibility	*The data is supplied with this article*

**Value of the data**

• The current paper illustrate the ability of exogenous GAPDH to cause apoptosis in SH-SY5Y cells.
• Apoptotic processes may be induced by GAPDH in different forms, including aggregates.
• The GAPDH binder hydrocortisone 21-hemisuccinate (RX624) has no effect on apoptosis-inducing GAPDH properties.

## 1 Data

The data presented in this article demonstrate that different fractions of pre-denatured GAPDH may cause apoptotic processes in neuroblastoma cells. We used the GAPDH binder RX624 to preserve cells from exogenous GAPDH [2], [3]. This paper contains data showing the ability of RX624 to affect the apoptosis induced by exogenous GAPDH.

## 2 Experimental design, materials, and methods

**Fig. 1:**
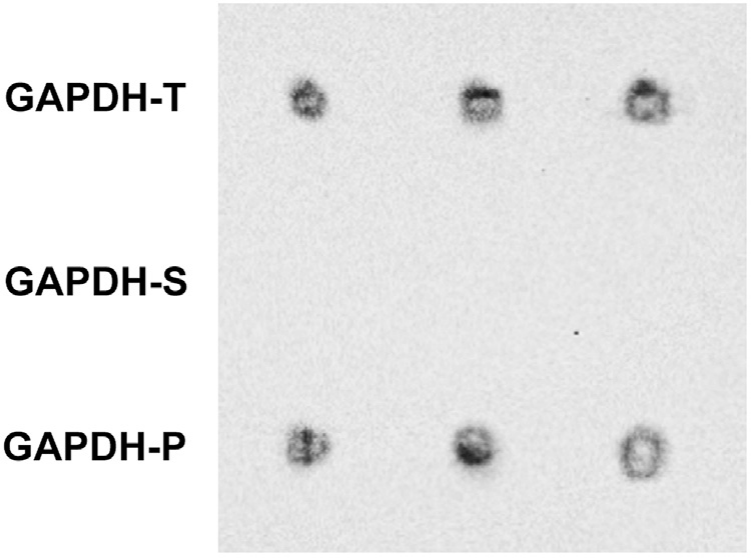
The data of ultrafiltration of different GAPDH fractions accompanied by a subsequent conjugation with anti-GAPDH 6C5 antibodies are shown.

**Fig. 2:**
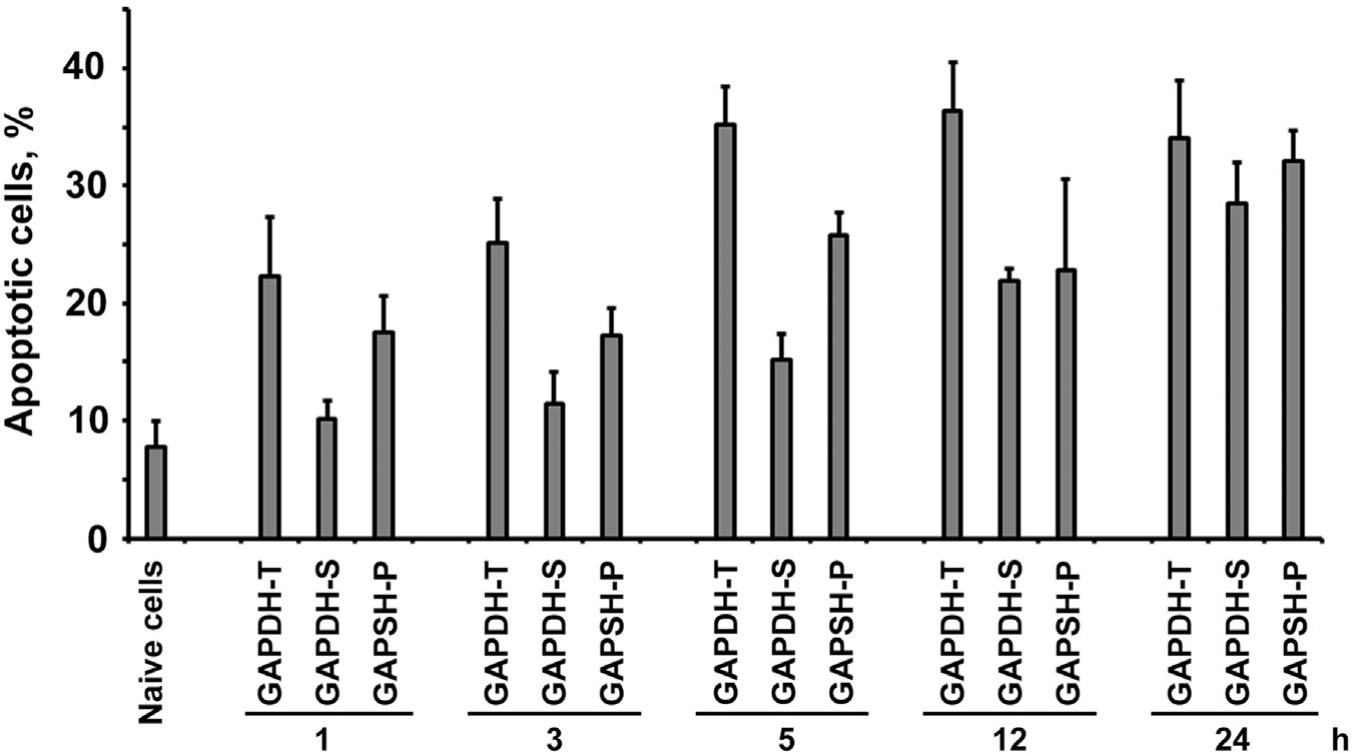
Different GAPDH fractions were incubated with SH-SY5Y cells for 1, 3, 5, 12 or 24 h. The proportion of apoptotic cells was measured using acridine orange staining.

**Fig. 3:**
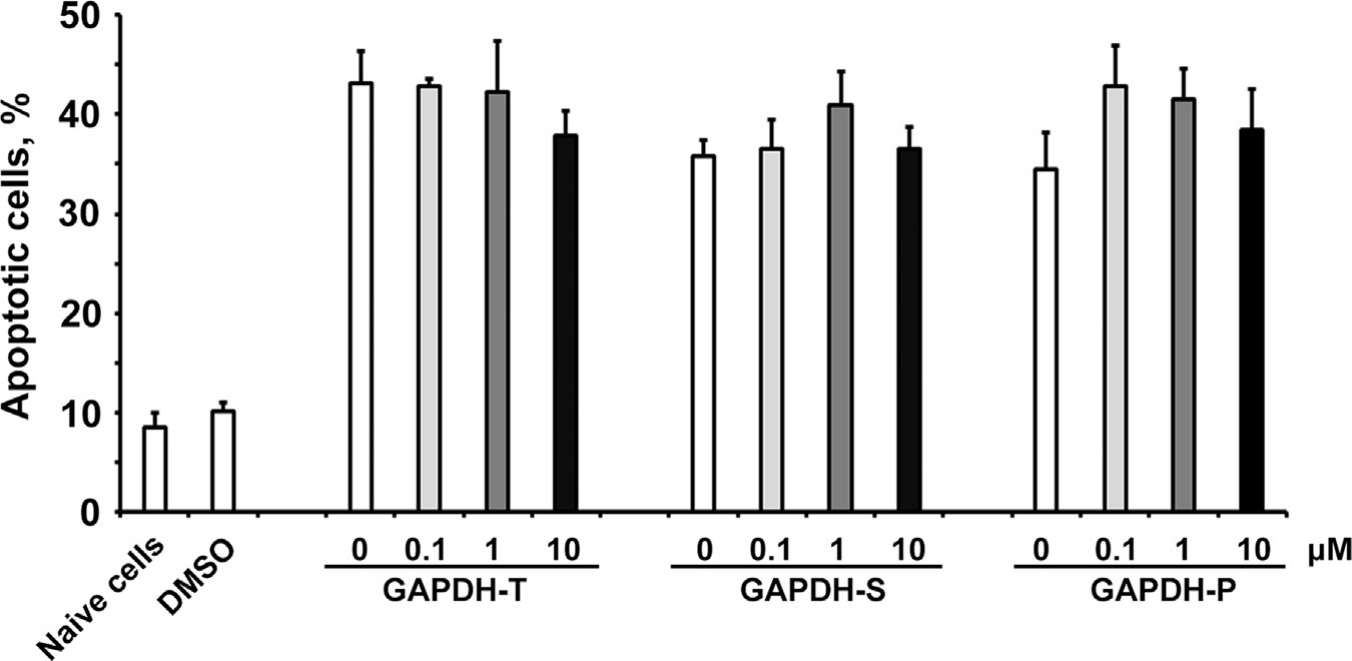
Different GAPDH fractions were incubated with SH-SY5Y for 24 h in the presence of various concentrations of RX624 (from 0.1 to 10 μM). The proportion of apoptotic cells was measured as in Fig. 1.

The procedure of the preliminary GAPDH denaturation and its separation into fractions as well as SH-SY5Y cell culturing conditions are described in detail in a related article [1]. Therefore, we used 3 GAPDH fractions: to verify the aggregate content in each fraction we applied ultrafiltration through acetate cellulose membrane (Fig. 1) as described earlier [4]. To explore the dynamics of apoptosis, the cells were placed in a 96-well micro-titre plate, and acridine orange in phosphate-buffered saline was added to a concentration of 5 μg/mL. Stained cells were analysed with the aid of a Zeiss Axioscope (Zeiss, Jena, Germany) (Fig. 2, Fig. 3).
